# Hepatitis B Virus Reactivation Associated With Therapeutic Interventions

**DOI:** 10.3389/fmed.2021.770124

**Published:** 2022-01-14

**Authors:** Young Chang, Soung Won Jeong, Jae Young Jang

**Affiliations:** Department of Internal Medicine, Institute for Digestive Research, Digestive Disease Center, Soonchunhyang University College of Medicine, Seoul, South Korea

**Keywords:** hepatitis B, reactivation, immunosuppressant, molecular target agents, direct-acting antivirals

## Abstract

Hepatitis B virus (HBV) reactivation associated with various therapeutic interventions is an important cause of morbidity and mortality in patients with current or resolved HBV infection. Because no curative treatment for HBV infection is yet available, there are many individuals at risk for HBV reactivation in the general population. Populations at risk for HBV reactivation include patients who are currently infected with HBV or who have been exposed to HBV in the past. HBV reactivation and its potential consequences is a concern when these populations are exposed to anti-cancer chemotherapy, immunosuppressive or immunomodulatory therapies for the management of various malignancies, rheumatologic diseases, inflammatory bowel disease, or solid-organ or hematologic stem cell transplantation. Accordingly, it has become important to understand the basics of HBV reactivation and the mechanisms by which certain therapies are more susceptible to HBV reactivation. This review aims to raise the awareness of HBV reactivation and to understand the mechanisms and the risks of HBV reactivation in various clinical settings.

## Introduction

Hepatitis B virus (HBV) infection is a global health concern. Worldwide, approximately two billion individuals have been infected with HBV at some point in their lifetime, resulting in seropositivity for anti-hepatitis B core antigen (anti-HBc) (1). Despite long-term control of HBV replication, current antiviral therapies are still unable to eradicate HBV in patients with chronic infection. HBV is known to be potentially persistent once it infects a host; however, the risk of reactivation is not always recognized by physicians when treating patients with comorbidities such as solid and hematologic malignancies, organ transplants, and rheumatoid and chronic inflammatory diseases. Although, HBV reactivation usually occurs in hepatitis B surface antigen (HBsAg)-positive patients, anti-HBc-positive patients who are HBsAg-negative can also experience HBV reactivation (2). HBV reactivation is not uncommon in patients receiving immunosuppressive therapy, which can lead to severe manifestations such as hepatitis flare, decompensation, and hepatic failure.

Over the past few years, the occurrence of HBV reactivation after therapeutic interventions has increased remarkably. B cell-depleting agents, such as rituximab, are known to be closely related to HBV reactivation, and corticosteroids are also frequently associated with HBV reactivation. In addition to anti-cancer drugs, immune modulating agents such as tumor necrosis factor (TNF) inhibitors, used to treat inflammatory bowel disease or rheumatologic diseases, can also induce HBV reactivation (3).

Prevention of HBV reactivation is necessary to reduce the risk of morbidity and mortality in patients receiving immunosuppressive or immunomodulatory agents. This review discusses the heterogeneous definitions of HBV reactivation, the risk of reactivation with a spectrum of various immunosuppressive and immunomodulatory agents, and recommendations for the prevention of HBV reactivation.

## Definition of HBV Reactivation

HBV reactivation refers to significant disturbances in the balanced state between the host immune system and viral replication. HBV reactivation may occur spontaneously or in response to therapeutic agents that affect the host immune system (4). Various definitions of HBV reactivation have been used in previous studies, most of which have been proposed in immunosuppressive settings (5–8). These proposed definitions consist of virological or serological criteria, or both. The updated American Association for the Study of Liver Diseases (AASLD) guidelines (9) defined HBV reactivation in HBsAg-positive patients when any of the following criteria is fulfilled: (1) at least 2 log (or 100-fold) increase in HBV DNA compared to the baseline level; (2) HBV DNA at least 3 log (or 1,000) IU/mL in patients with previously undetectable HBV DNA; or (3) HBV DNA at least 4 log (or 10,000) IU/mL if the baseline level is unavailable. The American Gastroenterological Association (AGA) guidelines (10) defined it stricter as (1) at least 10-fild increase in HBV DNA compared to the baseline or (2) *de novo* detectable DNA in patients with previously undetectable DNA. The European Association for the Study of the Liver (EASL) guidelines have not explicitly defined HBV reactivation, and the Asian-Pacific Association of the Study of the Liver (APASL) guidelines (11) defined it as (1) at least 100-fold increase in HBV DNA compared to the baseline or (2) at least 100 IU/mL inpatients with previously undetectable HBV DNA. In HBsAg-negative and anti-HBc-positive patients, detectable HBV DNA, reverse HBsAg seroconversion, or the reappearance of HBsAg are considered indicative of HBV reactivation.

The spectrum of patients with HBV reactivation is diverse. Some patients are asymptomatic with a normal hepatic biochemical profile, while others may have hepatitis flares, characterized by elevated alanine transaminase (ALT) levels of more than three times the baseline and >100 U/L. The severity of hepatitis flares can also vary from asymptomatic to hepatic decompensation, resulting in a poor prognosis.

## Mechanisms of HBV Reactivation

HBV infects individuals and leads to acute or chronic infections. Once HBV enters the hepatocytes, it releases a double-stranded viral genome that is transported into the nucleus. The viral genome is converted into plasmid-like covalently closed circular DNA (cccDNA) in the nucleus, forming a viral mini-chromosome (12). The cccDNA is stabilized in the hepatocytes and persists in a latent state, serving as a reservoir for HBV reactivation. Clinical recovery of HBV infection does not necessarily indicate a complete cure of HBV infection, as cccDNA and integrated viral DNA persist in the nucleus of hepatocytes (13). Theoretically, a complete cure of HBV infection is achieved by HBV cccDNA eradication, making viral replication impossible (14). However, complete eradication of both HBV cccDNA and integrated DNA is insufficient both in the host immune response and in antiviral treatment with nucleos(t)ide analogs. HBV reactivation is based on the presence of fully replication-competent cccDNA in the nucleus of infected hepatocytes. Even when only one copy of cccDNA remains in the hepatocytes, HBV replication can cause detectable viremia within a certain period of time upon HBV reactivation.

Based on the presence of cccDNA, HBV reactivation may occur in any situation, disrupting the balance between the host immune system and viral replication. The disruption can be brought about in two ways: immunosuppression mediated weakening of host immune control and increases in HBV viral replication (15). Any form of immunosuppression impairs host immune-mediated control of viral replication and can lead to HBV reactivation. Several immunosuppressive therapeutics, including B or T cell depleting agents, anticancer chemotherapeutic agents, corticosteroids, and various pathways of biologics, can cause host immune dysfunctions, resulting in the suppression of anti-HBV immunity. On the other hand, several treatments can have a direct impact on HBV replication. Corticosteroids not only suppress host immune control but also induce direct activation of HBV gene regulation (16), and mTOR inhibitors can suppress HBsAg synthesis at the transcriptional level through a feedback mechanism (17), thereby increasing HBV replication and reactivation. In addition, histone deacetylase inhibitors for cancer treatment have been reported to induce HBV reactivation by regulating cccDNA transcription-related histones (18).

## HBV Serologic Status and the Risk of HBV Reactivation

Various factors affecting reactivation of HBV is listed in Table 1. HBsAg-positive patients are at a higher risk for HBV reactivation than those with an HBsAg-negative and anti-HBc-positive serologic status. HBsAg-positive patients with hepatitis B e-antigen (HBeAg)-positive and/or high baseline HBV DNA levels >10,000 IU/mL are at greatest risk of HBV reactivation (19). Even if the HBV infection is resolved in HBsAg-negative and anti-HBc-positive patients, they are at risk for HBV reactivation when receiving immunosuppressive therapy, especially B cell-depleting agents (e.g., rituximab) or hematopoietic stem cell transplantation (21, 22).

**Table 1:** Factors affecting reactivation of hepatitis B.

Serologic status of chronic hepatitis B	Risk factors	HBsAg positivity
		HBeAg positivity
		High HBV DNA level (>10,000 IU/mL) (19)
		High anti-HBc level (≥6.41 IU/mL) (20)
	Protective factors	Anti-HBs positivity
		High anti-HBs level (>56.48 mIU/mL) (20)
Genetic and epidemiologic factors	Risk factor Protective factor	Male Genotype A HBV
Types of immunosuppressive therapy	High risk	High dose corticosteroids
		Anthracycline derivatives
		B cell-depleting agents
		Intensive chemotherapy for hematopoietic stem cell transplantation
	Moderate risk	Tumor necrosis factor-alpha inhibitors
		Tyrosine kinase inhibitors
	Unspecified but not negligible risk	Direct-acting antivirals for HCV

*HBsAg, hepatitis B surface antigen; HBeAg, hepatitis B e-antigen; HBV, hepatitis B virus; anti-HBc, anti-hepatitis B core antibody; anti-HBs, anti-hepatitis B surface antibody; HCV, hepatitis C virus*.

In addition, the presence of anti-HBs further decreased the risk of HBV reactivation in patients with resolved HBV infection. A prospective study of HBV reactivation in lymphoma patients with resolved HBV infection showed a doubled 2-year cumulative rate of reactivation in patients without anti-HBs (68.3%) compared to those with anti-HBs (34.4%) (21). Another prospective study showed that reactivation occurred in 23.5% of patients without anti-HBs whereas it occurred in only 7.8% of patients with anti-HBs (23). Baseline anti-HBs positivity has also been reported as a significant protective factor for HBV reactivation in patients with rheumatic diseases other than lymphoma (hazard ratio [HR] 0.14; 95% confidence interval [CI] 0.03–0.62) (24). Quantification of baseline anti-HBc/anti-HBs levels may predict reactivation risk in lymphoma patients with resolved HBV infection. Patients with high anti-HBc (≥6.41 IU/mL) and low anti-HBs (<56.48 mIU/mL) had a higher risk of reactivation (HR 17.29; 95% CI 3.92–76.30) (20). The presence of anti-HBs suggests a more potent humoral anti-HBV immunity, reflecting the extent of susceptibility of HBV specific adaptive immunity. Alternatively, it may reflect the quantity of cccDNA prepared for HBV reactivation (15).

HBV core-related antigen (HBcrAg) is made up of three related viral proteins, HBV core antigen, HBeAg, and a truncated 22 kDa pre-core protein. In addition to anti-HBc/HBs levels, the risk of HBV reactivation can also be predicted with serum HBcrAg (25). HBcrAg positivity was reported to have a three-fold increased risk of HBV reactivation (HR 2.94; 95% CI 1.43–6.07) in HBsAg-/anti-HBc+ patients who received rituximab or allogeneic hematopoietic stem cell transplantation (26). HBcrAg-positive patients showed a significantly higher HBV reactivation rate than HBcrAg-negative patients (71.8 vs. 31%, *p* = 0.002). HBV RNA, pre-genomic RNA contained in virions and released from infected hepatocytes, is also one of emerging biomarker candidates which can help identifying patients with transcriptionally active cccDNA who are more prone to HBV reactivation (27), but the role of HBV RNA in prediction of HBV reactivation after immunosuppressive therapies remains to be determined.

## Genetic and Epidemiologic Factors for HBV Reactivation

Patients infected with non-A HBV genotypes are more prone to reactivation than those with genotype A infection (28). Genotype A HBV exhibits slower replication kinetics than other genotypes (29), creating a weaker cellular response (30), resulting in enhanced likelihood of chronicity while reducing the risk of reactivation. Genetic changes in viruses may alter the risk of HBV reactivation. Core promoter and/or pre-core HBV mutants, as well as a high degree of S gene variability, known to impair HBsAg antigenicity, have been identified in reactivated HBV isolates (28, 31, 32). These HBV isolates might have evaded the anti-HBV immunity of the host and promoted the risk of reactivation.

Epidemiologic factors were demonstrated to be associated with HBV reactivation. The male sex is known to be an independent risk factor for HBV reactivation (33, 34), and a prospective study has reported a three-fold higher risk of HBV reactivation in men than in women (35). Although the molecular mechanism is largely unknown, the androgen signaling pathway has been reported to be associated with increased transcription and replication of HBV genes, which may promote the development of HBV infections by upregulating HBV RNA transcription and inflammatory cytokine levels (36).

## Types of Immunosuppressive Therapy and the Risk of HBV Reactivation

The intensity of immunosuppression depends primarily on the type, dose, and duration of immunosuppressive therapy, which vary depending on the indication and response to therapy. Some representative agents, from conventional immunosuppressive agents to molecular targeted agents, are highlighted below.

### Corticosteroids

Corticosteroids induce HBV reactivation by two mechanisms: suppression of cytotoxic T cell function and direct stimulation of the HBV genomic sequence (3). A retrospective study of HBsAg-positive patients with asthma or chronic obstructive pulmonary disease found that HBV reactivation occurred in 15.8% of patients treated with systemic corticosteroids continuously for 3 months, substantially higher than the 3.2% of patients treated with inhaled corticosteroids (odds ratio [OR] 5.72; 95% CI 1.17–27.91) (37). Among patients receiving systemic corticosteroids, a higher risk of reactivation (14.0%) was reported in those receiving corticosteroid doses >20 mg/day prednisolone or equivalent compared to those receiving low-dose systemic corticosteroid (4.5%) (37). Patients receiving low-dose corticosteroids, containing <10 mg/day prednisolone or equivalent, showed low risk of HBV reactivation even after prolonged use (10).

Treatment with a dosage of >20 mg/day of prednisolone, or its equivalent, and a duration longer than 2 weeks is generally considered clinically significant immunosuppression (38). Accordingly, a short period of corticosteroid therapy of <1 week was believed to have a low risk of causing HBV reactivation. However, a recent study found that the risk of hepatitis flare began to increase in chronic hepatitis B patients who received high peak doses of corticosteroid of >40 mg of prednisolone or equivalent, even after <7 days of administration (39). In patients receiving high-dose corticosteroid (>40 mg of prednisolone or equivalent), the risk of hepatitis flare was close to 10% (9.3–11.6%) for which antiviral prophylaxis was recommended, regardless of treatment duration (39).

### Anthracyclines and Conventional Chemotherapeutic Agents

Anthracyclines such as doxorubicin and epirubucin are widely used chemotherapeutic agents either alone or as a combination therapy in the treatment of breast cancer, ovarian cancer, lymphoma, and sarcoma. This drug class is a representative chemotherapeutic agent known to be associated with a significant risk of HBV reactivation (10). A prospective study found that the HBV reactivation rate was as high as 41% in HBsAg-positive patients receiving conventional chemotherapy for breast cancer, which had a significant impact on early termination or delay of the treatment schedule (40). Based on the results of that study, surveillance and prophylaxis of HBV infection has been widely practiced.

Concomitant use of immunosuppressive or immunomodulatory agents may increase the intensity of immunosuppression, resulting in an increased risk of HBV reactivation. A randomized controlled trial of HBsAg-positive patients receiving ACE (epirubicin, cyclophosphamide, and etoposide) or PACE (prednisolone plus ACE) chemotherapy found that a corticosteroid-free regimen had a significantly lower risk of HBV reactivation, with cumulative incidences of HBV reactivation of 38% and 73% for the ACE and PACE groups, respectively (relative risk 2.36; 95% CI 1.05–5.29) (41).

The highest risk of HBV reactivation has been reported in patients undergoing hematopoietic stem cell transplantation (HSCT), who typically receive intensive chemotherapy for the underlying malignancy to induce remission, followed by additional chemotherapy or radiation therapy for myeloablation. The HBV reactivation rate in patients with resolved HBV infection who received HSCT was 19.7%, and the 1-, 2-, and 4-year cumulative probabilities of reactivation were 9.0, 21.7, and 42.9%, respectively (42). Similar reactivation rates were reported in another study, with 1- and 5-year cumulative rates of 13% and 22%, respectively (43).

### Rituximab and Other B Cell-Depleting Agents

Rituximab is a monoclonal antibody against CD20 expressed on the surface of B lymphocytes, which targets and destroys B cells. It is used to treat hematologic malignancies and various inflammatory rheumatic diseases. B cells contribute to HBV clearance by producing neutralizing antibodies, preventing the spread of viruses, and eliminating circulating viruses. Although T lymphocytes-mediated immune control is thought to be the predominant mechanism by which HBV replication is inhibited (44), the compelling evidence of B cell immunity suppression, leading to HBV reactivation, suggests that B cells perform additional functions in suppressing HBV replication in addition to producing neutralizing antibodies (2).

The reported incidence of HBV reactivation after rituximab use was 3–55%, and the overall mortality rate from reactivation was reported to be as high as 30–38% (45). A recent prospective study of patients receiving obinutuzumab- or rituximab-containing immunochemotherapy showed HBV reactivation occurred in 8.2% of the patients, a median 125 days after the first dose (46). In patients without prophylactic antiviral therapy, 10.8% showed HBV reactivation, whereas in patients receiving prophylactic antiviral therapy, 2.1% showed HBV reactivation, suggesting that prophylactic antiviral therapy significantly reduced the risk of reactivation (adjusted HR 0.08, 95% CI 0.02–0.41) (46).

### Tumor Necrosis Factor-Alpha Inhibitors

TNF-α is a well-known proinflammatory agent, therefore biologics blocking TNF-α and related cytokine pathways have been used in various inflammatory and autoimmune diseases (47, 48). Its widespread use in various diseases has revealed the relevance of TNF-α to HBV reactivation. It was initially thought that these cytokines exert subtle regulation of the adaptive immune system responsible for HBV immune control, unlike other cytokines that induce non-specific inflammation. In particular, TNF-α can activate a unique host antiviral pathway, the APOBEC (apolipoprotein B mRNA editing enzyme, catalytic polypeptide-like) proteins, which cause the degradation of cccDNA in HBV-infected cells (49). Therefore, blocking this TNF-α-mediated antiviral pathway may cause HBV replication and reactivation.

A recent study of the incidence of HBV reactivation in patients treated with TNF-α inhibitor estimated a pooled incidence of HBV reactivation of 4.2% (95% CI 1.4–8.2%). The pooled incidence of reactivation in patients with resolved HBV infection was 3.0% (95% CI 0.6–7.2%) compared to 15.4% (95% CI 1.2–41.2%) in HBsAg-positive patients (50). Similarly, in a large retrospective study of patients receiving long-term TNF-α inhibitors, an HBV reactivation rate of 39% was reported in HBsAg-positive patients, whereas no reactivation occurred in patients with resolved HBV infection (6). In patients with resolved HBV infection, long-term biologic therapy using TNF-α inhibitors is not associated with an increased risk of HBV reactivation, and the risk of reactivation is negligibly low to none (51).

### Transarterial Chemoembolization

HBV reactivation have not so frequently reported with regard to transarterial chemoembolization (TACE) in patients with HBV-related HCC, as that of systemic chemotherapy. TACE is a kind of locoregional chemotherapy using Lipiodol which carry and localize chemotherapeutic agents inside the tumor (52). However, TACE and other local therapies for hepatocellular carcinoma can also contribute to HBV reactivation to different extents. Various results have been reported, showing a low risk of reactivation (53), to studies suggesting that reactivation occurs in 17.5–33.7% of patients with TACE, suggesting the association between TACE and reactivation of HBV (54, 55). The effect of TACE can be attributed mainly to the use of anthracyclines.

### Tyrosine Kinase Inhibitors

Activation of various kinase signaling pathways is essential for immune activation and lymphocyte proliferation (56). Many kinase inhibitors targeting these critical pathways have been developed to treat hematologic or other malignancies. Considering the importance of HBV-specific lymphocytes in the immune regulation of HBV replication, these kinase inhibitors may suppress the immune control of HBV replication, resulting in HBV reactivation. Several *in vitro* studies have shown that Bcr-Abl TKIs exert inhibitory effects on T cells (57), suggesting that T cell inhibition induced by Bcr-Abl TKIs may be a potential risk factor for HBV reactivation.

Several case reports and small-scale studies have suggested that tyrosine kinase inhibitors (TKIs), commonly used to treat chronic myeloid leukemia (CML) and gastrointestinal stromal tumors, may be associated with an increased risk of HBV reactivation (58–61). Two large-scale retrospective studies evaluating the risk of HBV reactivation in TKI-treated CML patients with positive HBsAg reported HBV reactivation rates of 26–34.8% (61, 62). More recently, a nationwide nested case-control study with 733,691 patients with HBV in Taiwan found that Bcr-Abl TKI use was independently associated with HBV reactivation (aHR, 1.56; 95% CI 1.11–2.20) (63).

### Calcineurin Inhibitors

Calcineurin inhibitors such as cyclosporine or tacrolimus suppress T cell function by inhibiting calcineurin required for signal transduction of T cell activation and inhibiting transcription of interleukin required for T cell proliferation (64). These immunosuppressive effects leading to broad immune dysfunctions could potentially lead to HBV reactivation. There have been several reports of HBV reactivation associated with the use of tacrolimus or cyclosporine in patients with rheumatic disease, some of which had serious consequences leading to death or liver transplantation (65, 66).

### Antimetabolites

There is no convincing evidence of HBV reactivation caused by antimetabolites monotherapy such as azathioprine, 6-mercaptopurine or methotrexate. Despite long-standing clinical use of azathioprine and its active metabolite, 6-mercaptopurin, there is no documented cases in which azathioprine or 6-mercaptopurin leads to HBV reactivation. Regarding methotrexate, a prospective study reported that HBV reactivation occurred in two of five patients with HBsAg and in one of 45 patients without HBsAg after methotrexate treatment in patients with rheumatoid arthritis (67). It is not certain whether methotrexate induced HBV reactivation, as this study included patients additionally receiving TNF-α to methotrexate. On the contrary, another cross-sectional study reported that no HBV reactivation was occurred with long-term use of methotrexate in rheumatologic patients (68).

### Direct-Acting Antivirals for Hepatitis C Virus

Although direct-acting antivirals (DAAs) are not considered immunosuppressive agents, DAAs targeting the hepatitis C virus (HCV) may result in HBV reactivation in patients co-infected with HBV and HCV. From 2013 to 2016, the U.S. Food and Drug Administration (FDA) identified 29 cases of H0BV reactivation in patients with HBV-HCV co-infection treated with DAAs, two of which resulted in death and one required liver transplantation (69). A recent meta-analysis of HBV reactivation in HBV-HCV co-infected patients showed that the estimated HBV reactivation rate in studies with interferon-free DAA-based therapy (12.2%) was not significantly different from interferon-based therapy (14.5%) (70). However, HBV reactivation was reported to occur much earlier with DAAs (4–12 weeks during treatment) than with interferon-based therapies (end of treatment or thereafter), and studies with DAA-based therapies were more likely to report hepatitis due to HBV reactivation (12.2% in DAAs vs. 0% in interferon) (70). Another meta-analysis demonstrated that HBV reactivation occurred in 24% of patients with positive HBsAg, compared to 1.4% in patients with resolved HBV infection after DAA treatment (8). Among the patients with resolved HBV infection, the titer of anti-HBs was reported to significantly decrease from the early stage of DAA treatment, suggesting patients with a negative or low titer of anti-HBs at baseline have potential risk of HBV reactivation (71).

In HBV-HCV co-infected patients, there are different viral dominance patterns, mostly HCV dominant, and the levels of HBsAg were found to be closely associated with viral dominance patterns (72). High levels of interferon γ-induced protein 10 (IP-10) were induced by HCV in HCV-dominant HBV-HCV co-infected patients, which is consistent with a low level of HBsAg (72). It can be hypothesized that HCV suppresses HBV DNA replication and HBsAg production by immune mechanisms such as the induction of IP-10. In these circumstances, HBV replication may be promoted when the immune mechanisms that suppress HBV replication are eliminated by DAA treatment for HCV infection.

### Immune Checkpoint Inhibitors

Immune checkpoint inhibitors target key regulators of immune system to suppress various kinds of tumors. Programmed cell death 1 (PD-1) and programmed death-ligand 1 (PD-L1) inhibitors are representative immunotherapies that prevent the escape mechanism of tumor cells and promote restoration of T cell function. Cytotoxic T-lymphocyte antigen-4 (CTLA-4) is a negative regulator of T cell activation, and inhibition of CTLA-4 induces T cell activation and immune surveillance against cancer cells. There are rare case reports of HBV reactivation associated with immune checkpoint inhibitors, and only reports related to nivolumab (73), pembrolizumab (74) and ipilimumab-nivolumab sequential treatment (75). Theoretically, increased T cell activity induced by immune checkpoint inhibitors may provide durable control of HBV. A pilot study reported that nivolumab treatment was well-tolerated and reduced HBsAg titer in most HBeAg-negative patients with suppressed HBV viral load (76).

### Immunosuppressive Therapies Used for SARS-CoV-2

There is a risk of HBV reactivation in severe SARS-CoV-2 infected patients. Disruption of the balance between the host's immune status and viral replication contributes to HBV reactivation after SARS-CoV-2 infection, and the intensity of immunosuppressive therapies is a major risk factor for HBV reactivation. HBV reactivation in patients infected with SARS-CoV-2 is usually associated with immunosuppressive therapy such as IL-6 or IL-1 receptor antagonists (tocilizumab, anakinra), and high-dose corticosteroids (77, 78). There are several reports of HBV reactivation in SARS-CoV-2 infected patients. A retrospective study found that three out of 21 patients with SARS-CoV-2 and HBV coinfection developed HBV reactivation, of which two received corticosteroid therapy (79). The most recent prospective study evaluated the risk of HBV reactivation in HBsAg-/anti-HBc+ patients with severe SARS-CoV-2 infection receiving immunosuppressive therapy (80). At 1-year follow-up, there were no cases of HBsAg seroconversion and two out of 69 had detectable serum HBV DNA, suggesting a low risk of HBV reactivation in patients with severe SARS-CoV-2 infection and resolved HBV infection.

## Management and Prophylaxis of HBV Reactivation

Immunosuppressed patients who develop HBV reactivation should be treated with antiviral agents with high efficacy and genetic barriers to resistance. Tenofovir or entecavir is the treatment of choice and tenofovir is preferred over entecavir in patients previously treated with lamivudine (81). Tenofovir and entecavir are recommended for prophylaxis as well, and no studies have yet investigated tenofovir alafenamide as a prophylactic agent. Although timely antiviral treatment can improve HBV flares in patients with HBV reactivation, some patients may progress to hepatic failure despite antiviral therapy (23). Therefore, it is important to start antiviral treatment early and to conduct prophylactic antiviral treatment in risk groups.

In terms of antiviral prophylaxis, tailored management based on risk stratification is essential to prevent HBV reactivation. HBV reactivation risk is categorized into high (>10%), moderate (1–10%), and low (<1%) (Table 2). All HBsAg-positive patients who are at moderate to high risk of HBV reactivation should be considered candidates for prophylactic antiviral therapy. In case of HBsAg-negative patients, antiviral prophylaxis is recommended in high risk group. It is generally recommended to start antiviral treatment before immunosuppressive treatment and continue it for 6–12 months after cessation of immunosuppression. Since a large proportion of reactivation cases after discontinuation of antiviral treatment have been reported, it is recommended to continue biochemical monitoring once antiviral prophylaxis is withdrawn (9, 82).

**Table 2:** HBV reactivation risk according to serologic status of chronic hepatitis B and immunosuppressive agents.

	**HBsAg+/anti-HBc+**	**HBsAg-/anti-HBc+**
High risk (>10%)	• B cell-depleting agents • Anthracycline derivatives • Corticosteroid therapy for ≥4 weeks (moderate to high dose)	• B cell-depleting agents
Moderate risk (1–10%)	• TNF-α inhibitors • Calcineurin inhibitors and other cytokine inhibitors • Tyrosine kinase inhibitors • Corticosteroid therapy for ≥4 weeks (low dose)	• Anthracycline derivatives • TNF-α inhibitors • Calcineurin inhibitors and other cytokine inhibitors • Tyrosine kinase inhibitors • Corticosteroid therapy for ≥4 weeks (moderate to high dose)
Low risk (<1%)	• Antimetabolites (traditional immunosuppressive agents) • Corticosteroid therapy for ≤ 1 week	• Corticosteroid therapy for ≥4 weeks (low dose) • Antimetabolites (traditional immunosuppressive agents) • Corticosteroid therapy for ≤ 1 week

*HBV, hepatitis B virus; HBsAg, hepatitis B surface antigen; anti-HBc, anti-hepatitis B core antibody; TNF-α, tumor necrosis factor-α*.

## Conclusion

HBV can persist as either an overt or occult infectious state without HBsAg once an infection has occurred. Since HBV persists within the nucleus of hepatocytes in the form of cccDNA and integrated DNA despite immune control, HBV has the potential to be reactivated spontaneously or under certain circumstances. HBV reactivation can result from any modulation of the virus or host immune system that interferes with the interaction between the virus and the host. In particular, the risk of HBV reactivation increases in patients receiving immunosuppressive therapy. Additionally, there are many novel agents that modulate the immune system, such as biologics and immunotherapeutics, which have the potential to induce HBV reactivation. Therefore, testing and monitoring HBV serology is recommended for patients who are candidates for immunosuppressive or immune-modulating treatment, and prompt initiation of antiviral therapy is needed when HBV reactivation is recognized.

## Author Contributions

JJ contributed to conception and design of the study. SJ organized the database. YC wrote the manuscript and revision. All authors contributed to manuscript revision, read, and approved the submitted version.

## Funding

This research was supported by Soonchunhyang University Research Fund.

## Conflict of Interest

The authors declare that the research was conducted in the absence of any commercial or financial relationships that could be construed as a potential conflict of interest.

## Publisher's Note

All claims expressed in this article are solely those of the authors and do not necessarily represent those of their affiliated organizations, or those of the publisher, the editors and the reviewers. Any product that may be evaluated in this article, or claim that may be made by its manufacturer, is not guaranteed or endorsed by the publisher.

## References

[1] Revill PA Chisari FV Block JM Dandri M Gehring AJ Guo H. A global scientific strategy to cure hepatitis B. Lancet Gastroenterol Hepatol. (2019) 4:545–58. 10.1016/S2468-1253(19)30119-0 30981686 PMC6732795

[2] Loomba R Liang TJ. Hepatitis B reactivation associated with immune suppressive and biological modifier therapies: current concepts, management strategies, and future directions. Gastroenterology. (2017) 152:1297–309. 10.1053/j.gastro.2017.02.009 28219691 PMC5501983

[3] Perrillo RP Gish R Falck-Ytter YT. American Gastroenterological Association Institute technical review on prevention and treatment of hepatitis B virus reactivation during immunosuppressive drug therapy. Gastroenterology. (2015) 148:221–44 e223. 10.1053/j.gastro.2014.10.038 25447852

[4] Wu T Li J Shao L Xin J Jiang L Zhou Q. Development of diagnostic criteria and a prognostic score for hepatitis B virus-related acute-on-chronic liver failure. Gut. (2018) 67:2181–91. 10.1136/gutjnl-2017-314641 28928275

[5] Paul S Saxena A Terrin N Viveiros K Balk EM Wong JB. Hepatitis B virus reactivation and prophylaxis during solid tumor chemotherapy: a systematic review and meta-analysis. Ann Intern Med. (2016) 164:30–40. 10.7326/M15-1121 26595058 PMC6410701

[6] Pauly MP Tucker LY Szpakowski JL Ready JB Baer D Hwang J. Incidence of hepatitis B virus reactivation and hepatotoxicity in patients receiving long-term treatment with tumor necrosis factor antagonists. Clin Gastroenterol Hepatol. (2018) 16:1964–73 e1961. 10.1016/j.cgh.2018.04.033 29702293

[7] Belperio PS Shahoumian TA Mole LA Backus LI. Evaluation of hepatitis B reactivation among 62,920 veterans treated with oral hepatitis C antivirals. Hepatology. (2017) 66:27–36. 10.1002/hep.29135 28240789

[8] Mucke MM Backus LI Mucke VT Coppola N Preda CM Yeh ML. Hepatitis B virus reactivation during direct-acting antiviral therapy for hepatitis C: a systematic review and meta-analysis. Lancet Gastroenterol Hepatol. (2018) 3:172–80. 10.1016/S2468-1253(18)30002-5 29371017

[9] Terrault NA Lok ASF McMahon BJ Chang KM Hwang JP Jonas MM. Update on prevention, diagnosis, and treatment of chronic hepatitis B: AASLD 2018 hepatitis B guidance. Hepatology. (2018) 67:1560–99. 10.1002/hep.29800 29405329 PMC5975958

[10] Reddy KR Beavers KL Hammond SP Lim JK Falck-Ytter YT American Gastroenterological Association I. American Gastroenterological Association Institute guideline on the prevention and treatment of hepatitis B virus reactivation during immunosuppressive drug therapy. Gastroenterology. (2015) 148:215–9. 10.1053/j.gastro.2014.10.039 25447850

[11] Sarin SK Kumar M Lau GK Abbas Z Chan HL Chen CJ. Asian-Pacific clinical practice guidelines on the management of hepatitis B: a 2015 update. Hepatol Int. (2016) 10:1–98. 10.1007/s12072-015-9675-4 26563120 PMC4722087

[12] Bock CT Schwinn S Locarnini S Fyfe J Manns MP Trautwein C. Structural organization of the hepatitis B virus minichromosome. J Mol Biol. (2001) 307:183–96. 10.1006/jmbi.2000.4481 11243813

[13] Werle-Lapostolle B Bowden S Locarnini S Wursthorn K Petersen J Lau G. Persistence of cccDNA during the natural history of chronic hepatitis B and decline during adefovir dipivoxil therapy. Gastroenterology. (2004) 126:1750–8. 10.1053/j.gastro.2004.03.018 15188170

[14] Lok AS Zoulim F Dusheiko G Ghany MG. Hepatitis B cure: from discovery to regulatory approval. J Hepatol. (2017) 67:847–61. 10.1016/j.jhep.2017.05.008 28778687

[15] Shi Y Zheng M. Hepatitis B virus persistence and reactivation. BMJ. (2020) 370:m2200. 10.1136/bmj.m2200 32873599

[16] Tur-Kaspa R Burk RD Shaul Y Shafritz DA. Hepatitis B virus DNA contains a glucocorticoid-responsive element. Proc Natl Acad Sci U S A. (1986) 83:1627–31. 10.1073/pnas.83.6.1627 3006059 PMC323136

[17] Teng CF Wu HC Tsai HW Shiah HS Huang W Su IJ. Novel feedback inhibition of surface antigen synthesis by mammalian target of rapamycin (mTOR) signal and its implication for hepatitis B virus tumorigenesis and therapy. Hepatology. (2011) 54:1199–207. 10.1002/hep.24529 21735472

[18] Pollicino T Belloni L Raffa G Pediconi N Squadrito G Raimondo G. Hepatitis B virus replication is regulated by the acetylation status of hepatitis B virus cccDNA-bound H3 and H4 histones. Gastroenterology. (2006) 130:823–37. 10.1053/j.gastro.2006.01.001 16530522

[19] Lau GK Leung YH Fong DY Au WY Kwong YL Lie A. High hepatitis B virus (HBV) DNA viral load as the most important risk factor for HBV reactivation in patients positive for HBV surface antigen undergoing autologous hematopoietic cell transplantation. Blood. (2002) 99:2324–30. 10.1182/blood.V99.7.2324 11895763

[20] Yang HC Tsou HH Pei SN Chang CS Chen JH Yao M. Quantification of HBV core antibodies may help predict HBV reactivation in patients with lymphoma and resolved HBV infection. J Hepatol. (2018) 69:286–92. 10.1016/j.jhep.2018.02.033 29551710

[21] Seto WK Chan TS Hwang YY Wong DK Fung J Liu KS. Hepatitis B reactivation in patients with previous hepatitis B virus exposure undergoing rituximab-containing chemotherapy for lymphoma: a prospective study. J Clin Oncol. (2014) 32:3736–43. 10.1200/JCO.2014.56.7081 25287829

[22] Seto WK Chan TS Hwang YY Wong DK Fung J Liu KS. Hepatitis B reactivation in occult viral carriers undergoing hematopoietic stem cell transplantation: a prospective study. Hepatology. (2017) 65:1451–61. 10.1002/hep.29022 28027590

[23] Hsu C Tsou HH Lin SJ Wang MC Yao M Hwang WL. Chemotherapy-induced hepatitis B reactivation in lymphoma patients with resolved HBV infection: a prospective study. Hepatology. (2014) 59:2092–100. 10.1002/hep.26718 24002804

[24] Chen YM Chen HH Huang WN Chen YH Hsieh TY Yang SS. Reactivation of hepatitis B virus infection following rituximab treatment in HBsAg-negative, HBcAb-positive rheumatoid arthritis patients: A long-term, real-world observation. Int J Rheum Dis. (2019) 22:1145–51. 10.1111/1756-185X.13582 31117160

[25] Mak LY Seto WK Fung J Yuen MF. New biomarkers of chronic hepatitis B. Gut Liver. (2019) 13:589–95. 10.5009/gnl18425 30919601 PMC6860035

[26] Seto WK Wong DK Chan TS Hwang YY Fung J Liu KS. Association of hepatitis B core-related antigen with hepatitis B virus reactivation in occult viral carriers undergoing high-risk immunosuppressive therapy. Am J Gastroenterol. (2016) 111:1788–95. 10.1038/ajg.2016.436 27644733

[27] Svicher V Salpini R Malagnino V Piermatteo L Alkhatib M Cerva C. New markers in monitoring the reactivation of hepatitis B virus infection in immunocompromised hosts. Viruses. (2019) 11:783. 10.3390/v11090783 31450680 PMC6784136

[28] Borentain P Colson P Coso D Bories E Charbonnier A Stoppa AM. Clinical and virological factors associated with hepatitis B virus reactivation in HBsAg-negative and anti-HBc antibodies-positive patients undergoing chemotherapy and/or autologous stem cell transplantation for cancer. J Viral Hepat. (2010) 17:807–15. 10.1111/j.1365-2893.2009.01239.x 20002298

[29] Sugiyama M Tanaka Y Kato T Orito E Ito K Acharya SK. Influence of hepatitis B virus genotypes on the intra- and extracellular expression of viral DNA and antigens. Hepatology. (2006) 44:915–24. 10.1002/hep.21345 17006908

[30] Ito K Yotsuyanagi H Yatsuhashi H Karino Y Takikawa Y Saito T. Risk factors for long-term persistence of serum hepatitis B surface antigen following acute hepatitis B virus infection in Japanese adults. Hepatology. (2014) 59:89–97. 10.1002/hep.26635 23897861

[31] Huang CH Yuan Q Chen PJ Zhang YL Chen CR Zheng QB. Influence of mutations in hepatitis B virus surface protein on viral antigenicity and phenotype in occult HBV strains from blood donors. J Hepatol. (2012) 57:720–9. 10.1016/j.jhep.2012.05.009 22634131

[32] Svicher V Cento V Bernassola M Neumann-Fraune M Van Hemert F Chen M. Novel HBsAg markers tightly correlate with occult HBV infection and strongly affect HBsAg detection. Antiviral Res. (2012) 93:86–93. 10.1016/j.antiviral.2011.10.022 22086128

[33] Yeo W Chan TC Leung NW Lam WY Mo FK Chu MT. Hepatitis B virus reactivation in lymphoma patients with prior resolved hepatitis B undergoing anticancer therapy with or without rituximab. J Clin Oncol. (2009) 27:605–11. 10.1200/JCO.2008.18.0182 19075267

[34] Yeo W Zee B Zhong S Chan PK Wong WL Ho WM. Comprehensive analysis of risk factors associating with Hepatitis B virus (HBV) reactivation in cancer patients undergoing cytotoxic chemotherapy. Br J Cancer. (2004) 90:1306–11. 10.1038/sj.bjc.6601699 15054446 PMC2409681

[35] Yeo W Chan PK Zhong S Ho WM Steinberg JL Tam JS. Frequency of hepatitis B virus reactivation in cancer patients undergoing cytotoxic chemotherapy: a prospective study of 626 patients with identification of risk factors. J Med Virol. (2000) 62:299–307. 10.1002/1096-9071(200011)62:3<299::aid-jmv1>3.0.co;2-0 11055239

[36] Zheng B Zhu YJ Wang HY Chen L. Gender disparity in hepatocellular carcinoma (HCC): multiple underlying mechanisms. Sci China Life Sci. (2017) 60:575–84. 10.1007/s11427-016-9043-9 28547581

[37] Kim TW Kim MN Kwon JW Kim KM Kim SH Kim W. Risk of hepatitis B virus reactivation in patients with asthma or chronic obstructive pulmonary disease treated with corticosteroids. Respirology. (2010) 15:1092–7. 10.1111/j.1440-1843.2010.01798.x 20630033

[38] Stuck AE Minder CE Frey FJ. Risk of infectious complications in patients taking glucocorticosteroids. Rev Infect Dis. (1989) 11:954–63. 10.1093/clinids/11.6.954 2690289

[39] Wong GL Yuen BW Chan HL Tse YK Yip TC Lam KL. Impact of dose and duration of corticosteroid on the risk of hepatitis flare in patients with chronic hepatitis B. Liver Int. (2019) 39:271–9. 10.1111/liv.13953 30179316

[40] Yeo W Chan PK Hui P Ho WM Lam KC Kwan WH. Hepatitis B virus reactivation in breast cancer patients receiving cytotoxic chemotherapy: a prospective study. J Med Virol. (2003) 70:553–61. 10.1002/jmv.10430 12794717

[41] Cheng AL Hsiung CA Su IJ Chen PJ Chang MC Tsao CJ. Steroid-free chemotherapy decreases risk of hepatitis B virus (HBV) reactivation in HBV-carriers with lymphoma. Hepatology. (2003) 37:1320–8. 10.1053/jhep.2003.50220 12774010

[42] Hammond SP Borchelt AM Ukomadu C Ho VT Baden LR Marty FM. Hepatitis B virus reactivation following allogeneic hematopoietic stem cell transplantation. Biol Blood Marrow Transplant. (2009) 15:1049–59. 10.1016/j.bbmt.2009.05.001 19660717

[43] Vigano M Vener C Lampertico P Annaloro C Pichoud C Zoulim F. Risk of hepatitis B surface antigen seroreversion after allogeneic hematopoietic SCT. Bone Marrow Transplant. (2011) 46:125–31. 10.1038/bmt.2010.70 20383209

[44] Guidotti LG Chisari FV. Immunobiology and pathogenesis of viral hepatitis. Annu Rev Pathol. (2006) 1:23–61. 10.1146/annurev.pathol.1.110304.100230 18039107

[45] Tsutsumi Y Yamamoto Y Ito S Ohigashi H Shiratori S Naruse H. Hepatitis B virus reactivation with a rituximab-containing regimen. World J Hepatol. (2015) 7:2344–51. 10.4254/wjh.v7.i21.2344 26413224 PMC4577642

[46] Kusumoto S Arcaini L Hong X Jin J Kim WS Kwong YL. Risk of HBV reactivation in patients with B-cell lymphomas receiving obinutuzumab or rituximab immunochemotherapy. Blood. (2019) 133:137–46. 10.1182/blood-2018-04-848044 30341058 PMC6337873

[47] Bandzar S Gupta S Platt MO. Crohn's disease: a review of treatment options and current research. Cell Immunol. (2013) 286:45–52. 10.1016/j.cellimm.2013.11.003 24321565

[48] Hwang YG Moreland LW. Induction therapy with combination TNF inhibitor and methotrexate in early rheumatoid arthritis. Curr Rheumatol Rep. (2014) 16:417. 10.1007/s11926-014-0417-8 24619653

[49] Lucifora J Xia Y Reisinger F Zhang K Stadler D Cheng X. Specific and nonhepatotoxic degradation of nuclear hepatitis B virus cccDNA. Science. (2014) 343:1221–8. 10.1126/science.1243462 24557838 PMC6309542

[50] Cantini F Boccia S Goletti D Iannone F Leoncini E Panic N. HBV reactivation in patients treated with antitumor necrosis factor-alpha (TNF-alpha) agents for rheumatic and dermatologic conditions: a systematic review and meta-analysis. Int J Rheumatol. (2014) 2014:926836. 10.1155/2014/926836 25114684 PMC4119686

[51] Barone M Notarnicola A Lopalco G Viggiani MT Sebastiani F Covelli M. Safety of long-term biologic therapy in rheumatologic patients with a previously resolved hepatitis B viral infection. Hepatology. (2015) 62:40–6. 10.1002/hep.27716 25613809

[52] Vogl TJ Naguib NN Nour-Eldin NE Rao P Emami AH Zangos S. Review on transarterial chemoembolization in hepatocellular carcinoma: palliative, combined, neoadjuvant, bridging, and symptomatic indications. Eur J Radiol. (2009) 72:505–16. 10.1016/j.ejrad.2008.08.007 18835117

[53] Park JW Park KW Cho SH Park HS Lee WJ Lee DH. Risk of hepatitis B exacerbation is low after transcatheter arterial chemoembolization therapy for patients with HBV-related hepatocellular carcinoma: report of a prospective study. Am J Gastroenterol. (2005) 100:2194–200. 10.1111/j.1572-0241.2005.00232.x 16181368

[54] Lao XM Luo G Ye LT Luo C Shi M Wang D. Effects of antiviral therapy on hepatitis B virus reactivation and liver function after resection or chemoembolization for hepatocellular carcinoma. Liver Int. (2013) 33:595–604. 10.1111/liv.12112 23402625

[55] Jang JW Choi JY Bae SH Kim CW Yoon SK Cho SH. Transarterial chemo-lipiodolization can reactivate hepatitis B virus replication in patients with hepatocellular carcinoma. J Hepatol. (2004) 41:427–35. 10.1016/j.jhep.2004.05.014 15336446

[56] Rowinsky EK. The erbB family: targets for therapeutic development against cancer and therapeutic strategies using monoclonal antibodies and tyrosine kinase inhibitors. Annu Rev Med. (2004) 55:433–57. 10.1146/annurev.med.55.091902.104433 14746530

[57] Cwynarski K Laylor R Macchiarulo E Goldman J Lombardi G Melo JV. Imatinib inhibits the activation and proliferation of normal T lymphocytes *in vitro*. Leukemia. (2004) 18:1332–9. 10.1038/sj.leu.2403401 15190258

[58] Lakhani S Davidson L Priebat DA Sherker AH. Reactivation of chronic hepatitis B infection related to imatinib mesylate therapy. Hepatol Int. (2008) 2:498–9. 10.1007/s12072-008-9099-5 19669326 PMC2716906

[59] Kang BW Lee SJ Moon JH Kim SN Chae YS Kim JG. Chronic myeloid leukemia patient manifesting fatal hepatitis B virus reactivation during treatment with imatinib rescued by liver transplantation: case report and literature review. Int J Hematol. (2009) 90:383–7. 10.1007/s12185-009-0386-2 19641858

[60] Lai GM Yan SL Chang CS Tsai CY. Hepatitis B reactivation in chronic myeloid leukemia patients receiving tyrosine kinase inhibitor. World J Gastroenterol. (2013) 19:1318–21. 10.3748/wjg.v19.i8.1318 23483799 PMC3587491

[61] Uhm J Kim S-H Oh S Zang DY Do YR Lee WS. High incidence of hepatitis B viral reactivation in chronic myeloid leukemia patients treated with tyrosine kinase inhibitors. Blood. (2018) 132:3010–3010. 10.1182/blood-2018-99-11754316462024

[62] Kim S-H Kim HJ Kwak J-Y Kim JS Mun Y-C Park JS. Hepatitis B virus reactivation in chronic myeloid leukemia treated with various tyrosine kinase inhibitors: multicenter, retrospective study. Blood. (2012) 120:3738–3738. 10.1182/blood.V120.21.3738.373816462024

[63] Wang LY Chu SC Lo Y Yang YY Chan KA. Association of Bcr-Abl tyrosine kinase inhibitors with hepatitis B virus reactivation requiring antiviral treatment in Taiwan. JAMA Netw Open. (2021) 4:e214132. 10.1001/jamanetworkopen.2021.4132 33822067 PMC8025118

[64] Azzi JR Sayegh MH Mallat SG. Calcineurin inhibitors: 40 years later, can't live without. J Immunol. (2013) 191:5785–91. 10.4049/jimmunol.1390055 24319282

[65] Hagiyama H Kubota T Komano Y Kurosaki M Watanabe M Miyasaka N. Fulminant hepatitis in an asymptomatic chronic carrier of hepatitis B virus mutant after withdrawal of low-dose methotrexate therapy for rheumatoid arthritis. Clin Exp Rheumatol. (2004) 22:375–6. 15144137

[66] Ito S Nakazono K Murasawa A Mita Y Hata K Saito N. Development of fulminant hepatitis B (precore variant mutant type) after the discontinuation of low-dose methotrexate therapy in a rheumatoid arthritis patient. Arthritis Rheum. (2001) 44:339–42.. 10.1002/1529-0131(200102)44:2<339::AID-ANR51>3.0.CO;2-Q 11229464

[67] Tamori A Koike T Goto H Wakitani S Tada M Morikawa H. Prospective study of reactivation of hepatitis B virus in patients with rheumatoid arthritis who received immunosuppressive therapy: evaluation of both HBsAg-positive and HBsAg-negative cohorts. J Gastroenterol. (2011) 46:556–64. 10.1007/s00535-010-0367-5 21246383

[68] Laohapand C Arromdee E Tanwandee T. Long-term use of methotrexate does not result in hepatitis B reactivation in rheumatologic patients. Hepatol Int. (2015) 9:202–8. 10.1007/s12072-014-9597-6 25788188

[69] Bersoff-Matcha SJ Cao K Jason M Ajao A Jones SC Meyer T. Hepatitis B virus reactivation associated with direct-acting antiviral therapy for chronic hepatitis c virus: a review of cases reported to the U. S Food and Drug Administration Adverse Event Reporting System. Ann Intern Med. (2017) 166:792–8. 10.7326/M17-0377 28437794

[70] Chen G Wang C Chen J Ji D Wang Y Wu V. Hepatitis B reactivation in hepatitis B and C coinfected patients treated with antiviral agents: a systematic review and meta-analysis. Hepatology. (2017) 66:13–26. 10.1002/hep.29109 28195337

[71] Ogawa E Furusyo N Murata M Toyoda K Hayashi T Ura K. Potential risk of HBV reactivation in patients with resolved HBV infection undergoing direct-acting antiviral treatment for HCV. Liver Int. (2018) 38:76–83. 10.1111/liv.13496 28618152

[72] Wiegand SB Jaroszewicz J Potthoff A Honer Zu Siederdissen C Maasoumy B Deterding K. Dominance of hepatitis C virus (HCV) is associated with lower quantitative hepatitis B surface antigen and higher serum interferon-gamma-induced protein 10 levels in HBV/HCV-coinfected patients. Clin Microbiol Infect. (2015) 21:710 e711–9. 10.1016/j.cmi.2015.03.003 25882360

[73] Lake AC. Hepatitis B reactivation in a long-term nonprogressor due to nivolumab therapy. AIDS. (2017) 31:2115–8. 10.1097/QAD.0000000000001599 28906278

[74] Pandey A Ezemenari S Liaukovich M Richard I Boris A. A Rare case of pembrolizumab-induced reactivation of hepatitis B. Case Rep Oncol Med. (2018) 2018:5985131. 10.1155/2018/5985131 30416833 PMC6207901

[75] Koksal AS Toka B Eminler AT Hacibekiroglu I Uslan MI Parlak E. HBV-related acute hepatitis due to immune checkpoint inhibitors in a patient with malignant melanoma. Ann Oncol. (2017) 28:3103–4. 10.1093/annonc/mdx502 28945827

[76] Gane E Verdon DJ Brooks AE Gaggar A Nguyen AH Subramanian GM. Anti-PD-1 blockade with nivolumab with and without therapeutic vaccination for virally suppressed chronic hepatitis B: a pilot study. J Hepatol. (2019) 71:900–7. 10.1016/j.jhep.2019.06.028 31306680

[77] Chen LF Mo YQ Jing J Ma JD Zheng DH Dai L. Short-course tocilizumab increases risk of hepatitis B virus reactivation in patients with rheumatoid arthritis: a prospective clinical observation. Int J Rheum Dis. (2017) 20:859–69. 10.1111/1756-185X.13010 28160426

[78] Carroll MB. The impact of biologic response modifiers on hepatitis B virus infection. Expert Opin Biol Ther. (2011) 11:533–44. 10.1517/14712598.2011.554810 21269234

[79] Liu J Wang T Cai Q Sun L Huang D Zhou G. Longitudinal changes of liver function and hepatitis B reactivation in COVID-19 patients with pre-existing chronic hepatitis B virus infection. Hepatol Res. (2020) 50:1211–21. 10.1111/hepr.13553 32761993 PMC7436737

[80] Rodriguez-Tajes S Miralpeix A Costa J Lopez-Sune E Laguno M Pocurull A. Low risk of hepatitis B reactivation in patients with severe COVID-19 who receive immunosuppressive therapy. J Viral Hepat. (2021) 28:89–94. 10.1111/jvh.13410 32969557 PMC7537127

[81] Ekpanyapong S Reddy KR. Hepatitis B virus reactivation: what is the issue, and how should it be managed? Clin Liver Dis. (2020) 24:317–33. 10.1016/j.cld.2020.04.002 32620274

[82] European Association for the Study of the Liver. Electronic address EEE, European Association for the Study of the L. EASL 2017 Clinical Practice Guidelines on the management of hepatitis B virus infection. J Hepatol. (2017) 67:370–98. 10.1016/j.jhep.2017.03.021 28427875

